# Performance Evaluation of Ultra-Violet Light and Iron Oxide Nanoparticles for the Treatment of Synthetic Petroleum Wastewater: Kinetics of COD Removal

**DOI:** 10.3390/ma14175012

**Published:** 2021-09-02

**Authors:** Cecilia O. Akintayo, Omolola H. Aremu, Wilfred N. Igboama, Simphiwe M. Nelana, Olushola S. Ayanda

**Affiliations:** 1 Nanoscience Research Unit, Department of Industrial Chemistry, Federal University Oye Ekiti, P.M.B 373, Oye Ekiti 362001, Nigeria; cecilia.akintayo@fuoye.ng (C.O.A.); hellenlol@yahoo.com (O.H.A.); 2 Department of Physics, Federal University Oye Ekiti, P.M.B 373, Oye Ekiti 362001, Nigeria; wnigboama@gmail.com; 3 Department of Chemistry, Vaal University of Technology, Vanderbijlpark 1900, South Africa; simphiwen@vut.ac.za

**Keywords:** chemical oxygen demand, iron oxide nanoparticles, ultra-violet light, photocatalysis, refinery wastewater

## Abstract

In this study, the use of ultra-violet (UV) light with or without iron oxide nanoparticles (IONPs) for the degradation of synthetic petroleum wastewater was investigated. The IONPs was synthesised by sodium borohydride reduction of ferric chloride solution and was characterised by scanning electron microscopy (SEM), transmission electron microscopy (TEM), Fourier transform infrared spectrometry (FTIR), x-ray fluorescence spectrophotometry (XRF), and energy dispersive spectroscopy (EDS). The amount of degradation was evaluated by chemical oxygen demand (COD) determination. Experimental results show that the COD removal from synthetic petroleum wastewater by IONPs/UV system was more effective than they were independently. The combination of UV light at a wavelength of 254 nm, pH of 8, and 1.0 g of IONPs resulted in COD removal from 10.5% up to 95.5%. The photocatalytic degradation of synthetic petroleum wastewater is about 1.3–2.0 times faster in comparison to UV light only. The removal of COD from synthetic petroleum wastewater by UV light and IONPs follows the pseudo-first-order kinetic model with rate constant *k* ranging from 0.0133 min^−1^ to 0.0269 min^−1^. Consequently, this study has shown that the use of UV light in the presence of IONPs is favourable and effective for the removal of organic pollutants from petroleum refinery wastewater.

## 1. Introduction

Petroleum refineries are complex systems of multiple operations [1] that depend on the type of crude refined, the desired products, composition of condensate, and the treatment processes; therefore, the characteristics of refinery wastewater vary according to these complex patterns [2,3]. Many of the processes in petroleum refineries use a very large amount of water [4]. The refineries consequently generate a significant amount of wastewater that has been in contact with hydrocarbons, heavy metals and toxic compounds [5,6]. Water that is generated in the process units falls in the following categories: desalter effluent (resulting from desalting crude oil), sour water (steam condensed as an aqueous phase and removed as sour water), tank bottom draws (found in crude tanks, gasoline tanks, and slop tanks), and spent caustic (residual H_2_S, phenols, organic acids, hydrogen cyanide, and carbon dioxide). Refinery wastewater contains high levels of biochemical oxygen demand (BOD), chemical oxygen demand (COD), phenol, oil, benzene, benzo(a)pyrene, heavy metals, and other pollutants [7]. Refineries also generate solid wastes and sludges, 80% of which may be considered hazardous because of the presence of toxic organics and heavy metals [8,9]. Wastewater is typically treated in treatment plants and can sometimes be reused [5], sometimes requiring additional treatment to remove suspended solids and other contaminants. Tertiary treatments are considered if the refinery needs to meet stringent limits for different contaminants such as total suspended solids (TSS), COD, dissolved and suspended metals, and trace organics such as polyaromatic hydrocarbons (PAHs). The methods used in tertiary treatments include sand filtration, activated carbon, chemical oxidation, ion exchange, etc. Petroleum refineries discharge a large amount of wastewater that contains potentially toxic compounds into the environment, and these compounds are also difficult to degrade. Therefore, the use of the advanced oxidation processes (AOPs) will serve as a new feasible method for treating petroleum process wastewater for reuse and/or before the wastewater is discharge into the environment.

The AOPs have been reported to have widespread application of treating organic and inorganic pollutants present in water and wastewater [10,11,12]. These processes oxidize the complex organic pollutants found to be difficult to degrade biologically into simpler end products or degrades them to CO_2_ and H_2_O. AOP has been effectively utilized as a pre-treatment technique to remove toxic pollutants that interfere with or escape past wastewater treatment processes [13]. One of the specific features of this technique is the ability to completely mineralize organic contaminants without any trace of harmful residues. Complete degradation and mineralization potential of AOP may be due to the generation of very reactive hydroxyl and other radicals [14,15].

Photolysis, one of the categories of AOPs involves the degradation of the target compounds through the action of solar or ultra-violet (UV) light, with eventual formation of intermediate compounds which later break down to give the harmless end-products [16,17]. The technique is widely used to disinfect drinking water due to the availability of natural and UV light. Sanches et al. [18] explored low pressure UV photolysis to degrade priority pollutants such as pesticides present in water and in their study the photolysis process mineralized the target pollutants effectively. In a related finding by Sanches et al. [18], the combined treatment using hydrogen peroxide (H_2_O_2_), ozone (O_3_), and heterogeneous photocatalyst was applied to highly turbid water and the decomposition of the target pollutants was faster than UV treatment alone. Similarly, recent studies have demonstrated the efficacy of nano zero valent iron particles for the transformation of halogenated organic contaminants and heavy metals [19]. In these studies, wastewaters were treated with nanoparticles and this has made it possible to clean the polluted water with very little waste generation [20]. Several authors have reported the use of metal oxide nanoparticles such as TiO_2_ [21,22,23], ZnO [24], and zeolite [23] in AOPs for treating petroleum wastewater. The report on the use of iron oxide nanoparticles (IONPs) is limited.

Iron is a transition element which belongs to the d-orbital block elements because its valence shell electrons are in the d-orbital. Iron is the fourth most abundant element in the world. The properties of bulk iron are different from nano iron because of their enhanced surface area and small particle size. Nano iron has a large surface area resulting from its small particle size, which in turn makes the nanoparticles highly reactive. Nano iron can be obtained from different iron containing sources such as rock, waste tailing sand, and commercial chemicals [25,26]. Nano iron particles can be synthesized using different methods such as chemical precipitation, sol gel hydrolysis, electrochemical techniques, aerosol techniques, microemulsion methods, ultrasonic techniques, surfactant mediated techniques, etc [27]. Sodium borohydride reduction of ferric chloride solution was used for the synthesis of iron oxide nanoparticles (IONPs) in this study. Iron nanoparticles are cheap and have advantages over conventional treatment methods, which include less waste generation, an ability to treat both inorganic and organic contaminants, as well as neutralizing the smell of sulphide compounds. Studies have also shown that nano zero valent iron is highly reactive, effective, and compatible with other treatments and results in the production of non-hazardous end products [28]. Therefore, a combined UV light and IONPs treatment system will aid as a novel treatment method for petroleum wastewater. 

Petrochemical industries are major polluters of the environment; this has propelled the government to establish regulatory bodies saddled with the responsibility to check and monitor the activities of operators to reduce the rate of environmental pollution. The cost of treating wastewater generated at refineries is very high and has become a financial burden to the petrochemical industries, which eventually results in neglect that creates a continuing environmental problem. The current volume of refinery wastewater discharged into the sea amounts to million cubic meters per annum [2]. This substantial amount of wastewater could be recycled for various applications. To achieve this goal, the current refinery treatment processes should undergo significant upgrades and should include the advanced oxidation treatment unit.

In this study, the efficiency and kinetics of UV light and IONPs for the degradation of simulated petroleum wastewater was investigated. The effects of time, IONPs dosage, and pH were considered.

## 2. Materials and Methods

### 2.1. Chemicals and Reagents

All the reagents used in this work were of analytical grade and were used without further purification. Ferric chloride (FeCl_3_·6H_2_O) and sodium borohydride (NaBH_4_) used for the synthesis of IONPs were procured from Sigma-Aldrich (Diegem, Belgium). Diesel was purchased from a nearby petrol pump (Oye Ekiti, Nigeria). Phenol (C_6_H_5_OH), sodium sulphide (Na_2_S*x*H_2_O), ammonium chloride (NH_4_Cl), potassium nitrate (KNO_3_), and trace metal (MgSO_4_·7H_2_O, CaCl_2_·2H_2_O, FeCl_3_·6H_2_O, CuCl_2_, ZnCl_2_, NiCl_2_·6H_2_O, and CoCl_2_) solutions for the simulation of petroleum wastewater were obtained from Merck (Darmstadt, Germany). For studies on the effect of pH, 0.1 M HCl or NaOH were used for the adjustment of pH.

### 2.2. Instrumentation

Analyses such as the scanning electron microscopy (TESCAN MIRA3 SEM, Brno-Kohoutovice, Czech Republic), transmission electron microscopy (Tecnai G2 20 TEM, FEI, Hillsboro, OR, USA), X-ray fluorescence spectrophotometry (Phillips-JEE 4B, Worcestershire, UK), energy dispersive spectroscopy (EDS, attached to SEM), and Fourier transform infrared spectrometer FT-IR (Nicolet iS10 FT-IR Spectrometer, Thermo Scientific, MA, USA) was used to determine the specifications of the synthesised IONPs. The photocatalytic process was carried out with a UV lamp (UVP UVGL–15; 4 Watt, 230 V–50/60 Hz, Analytic, Jena, Germany).

### 2.3. Simulation of Petroleum Wastewater

The preparation of synthetic petroleum wastewater is similar to the method by Chakraborty and Veeramani [29]. The synthetic feed consisting of diesel, C_6_H_5_OH, Na_2_S*x*H_2_O, NH_4_Cl, KNO_3_, and phosphate buffer was prepared. The composition of stock trace metal solution was 10,000 mg/L MgSO_4_·7H_2_O, 10,000 mg/L CaCl_2_·2H_2_O, 5000 mg/L FeCl_3_·6H_2_O, 1000 mg/L CuCl_2_, 1000 mg/L ZnCl_2_, 500 mg/L NiCl_2_·6H_2_O, and 500 mg/L CoCl_2_. Deionised water was used in the preparation of the feed and trace metal solution. The resultant COD of the synthetic wastewater was 1200 mg/L, a characteristic property of a real petroleum refinery wastewater [30,31]

### 2.4. Synthesis of Iron Oxide Nanoparticles 

A method by Gilbert et al. [32], was adopted for the synthesis of IONPs. A total of 27 mL of 0.2 M FeCl_3_·6H_2_O was transferred into a 500 mL three-neck round bottom flask and titrated with 50 mL of 0.2 M NaBH_4_ solution; the solution was stirred vigorously with a stirring rod until all the NaBH_4_ solution was completed. The suspended black IONPs was filtered with Whatmann No. 32 filter paper and put in the oven at 333 K for 24 h.

### 2.5. Photocatalytic Degradation

**Figure 1:**
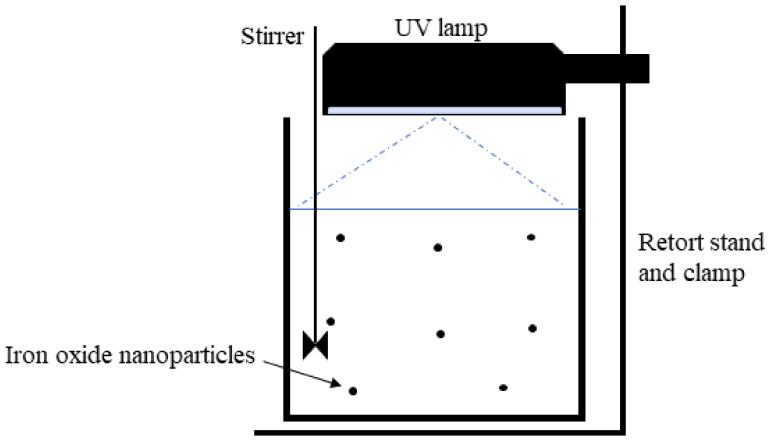
Schematic diagram of photocatalytic degradation.

The removal of COD from synthetic petroleum wastewater was carried out in the dark under irradiation of UV light (254 nm) in a reactor with or without IONPs. A scheme of the degradation is presented in Figure 1.

During photocatalysis, 0.2 g–1.0 g of the IONPs was added to 50 mL of synthetic petroleum wastewater. The UV lamp was localized above the reactor containing the solution, the distance between the UV lamp to the wastewater was about 14 cm. Samples were taken and filtered after the time has elapsed. The level of pollution was quantified by measurement of COD before and after the treatment processes, hence, the degradation efficiency (%) was obtained using Equation (1).

(1) $$\%\;\mathrm{Degradation}=\frac{{\mathrm{COD}}_{o}-{\mathrm{COD}}_{t}}{{\mathrm{COD}}_{o}}$$

where COD_o_ and COD_t_ are the initial and final COD, respectively.

### 2.6. COD Determination

COD describes the amount of oxygen required to chemically breakdown pollutants. It can be used to estimate the amount of pollution in water sample. The COD determination was conducted using the method by Adewoye et al. [33]. A total of 10 mL of 0.25 N potassium dichromate (K_2_Cr_2_O_7_), 30 mL of sulphuric acid, and Silva-sulphate (H_2_SO_4_ + Ag_2_SO_4_) were added to a COD vial containing 20 mL of the water sample. The solution was digested for 2 h in a COD digester. After digestion, the sample was cooled to ambient temperature and diluted to 150 mL with distilled water and the excess K_2_Cr_2_O_7_ that remained was titrated with ferrous ammonium sulphate using ferroin indicator. The concentration of COD in the water sample was determined by using Equation (2).

(2) $$\mathrm{COD}=\frac{\left(A-B\right)\times \;N\;\times 1000\times 8}{V}$$

where A is the volume (mL) of ferrous ammonium sulphate used for the blank, B is the volume (mL) of ferrous ammonium sulphate used for the water sample, N is the normality of ferrous ammonium sulphate, 8 is the milliequivalents of oxygen, and V is the volume of the water sample.

## 3. Results and Discussion

### 3.1. Characterization of Iron Oxide Nanoparticles 

**Figure 2:**
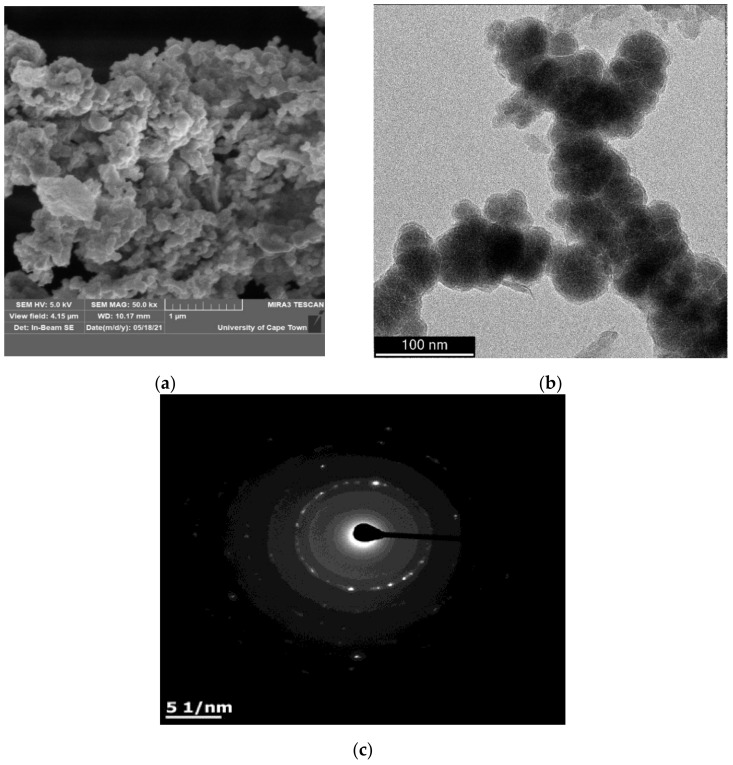
SEM (**a**), TEM (**b**), and SAED (**c**) of iron oxide nanoparticles.

**Figure 3:**
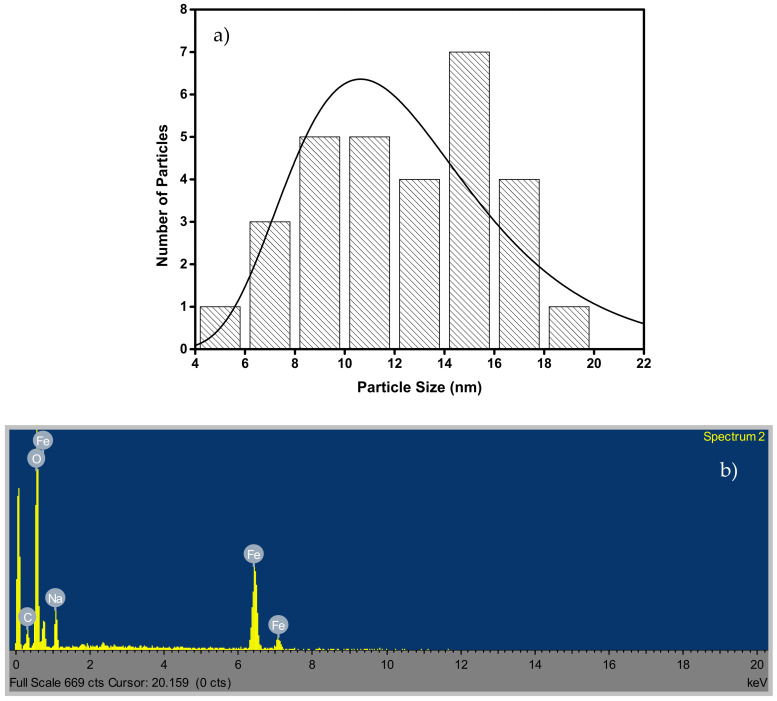
Particle size distribution (**a**) and EDX (**b**) of iron oxide nanoparticles.

The SEM of the IONPs at 50.0 kx magnification (Figure 2a) shows agglomerated nanosphere structure while the TEM micrograph (Figure 2b) confirms the regular spherical structures, which are connected to each other [34]. Therefore, the morphological studies confirmed that the synthesized IONPs yielded spherical bead-like structures attached to one another in a thread-like manner known as nanonecklace. The ring-type patterns of the selective area electron diffraction (SAED) of IONPs (Figure 2c) show that the structure was polycrystalline iron core with an amorphous iron oxide shell. The TEM micrograph was subjected to ImageJ 1.46r software for particle size distribution. The software was used to measure the diameter of particles in the material. The analysis shows that the diameter of the particles ranges between 4.6 and 19.8 nm (Figure 3a), and the mean particle size of the IONPs is 12.5 nm.

**Figure 4:**
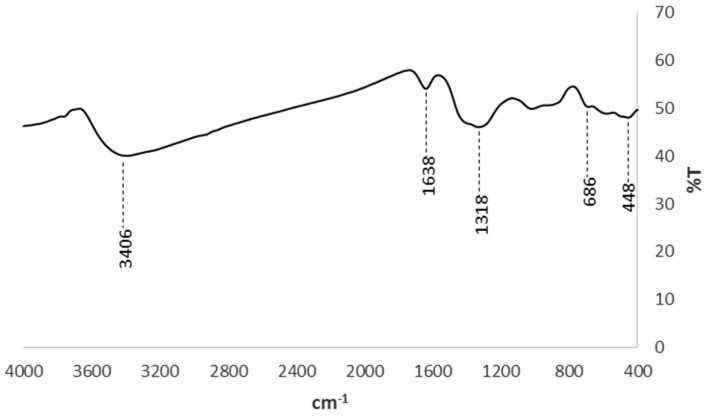
FTIR of iron oxide nanoparticles.

The EDS spectrum (Figure 3b) indicates the presence of Fe, C, O and Na. The presence of Na results from the precursor (NaBH_4_) used during synthesis. Moreover, the elemental composition of the IONPs is Fe (36.5%), C (12.3%), O (45.2%), and Na (6.0%). The IR spectrum of the synthesized IONPs is shown in Figure 4. The broad absorption band at 3406 cm^−1^ is the characteristic stretching vibration of –OH group, which is a function of the water adsorbed during synthesis [35]. The band at 1638 cm^−1^ may be ascribed to OH bending vibration and the characteristic peak 448 cm^−1^ and 686 cm^−1^ is assigned to the stretching vibration of Fe-O band.

Table 1 represents the chemical composition of the IONPs in weight percent. The species with the highest composition is Fe_2_O_3_, followed by SiO_2_. Thus, Fe_2_O_3_ is the major element of the synthesised IONPs.

### 3.2. Degradation Efficiency

#### 3.2.1. Effect of Time and Iron Oxide Nanoparticles Dosage

**Figure 5:**
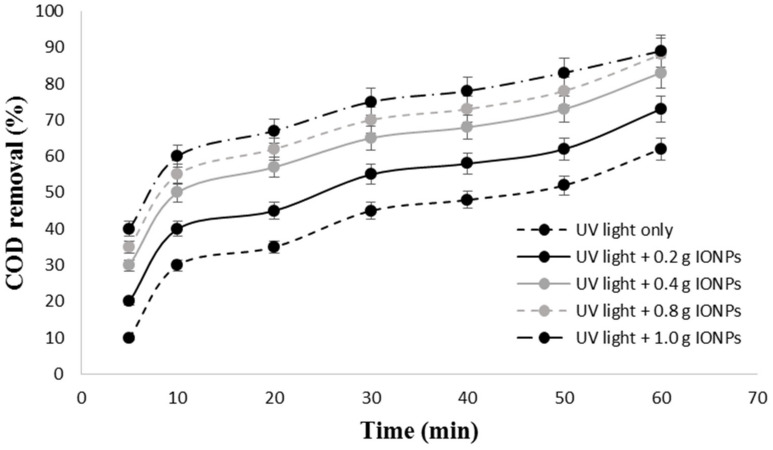
Effect of time and iron oxide nanoparticles dosage on COD removal from synthetic petroleum wastewater.

The effect of time and IONPs dosage on COD removal is depicted in Figure 5. The figure showed that the UV light only resulted to COD removal from 10.5% (5 min) to 62.2% (60 min), whereas an increase in IONPs dosage results in a very rapid increase in COD removal. The highest degradation efficiency with photocatalysis (UV light + IONPs) when compared to UV light only might be due to the synergistic effect between UV light and IONPs. The lower photocatalytic reaction rate at IONPs dosage of 0.2 g is due to less availability of active sites. The photocatalytic degradation of COD tends to increase as the catalyst loading increases. At IONPs dosage of 0.2 g the photocatalytic degradation of COD achieved at 60 min was 73.5%. The increase of the dosage to 1.0 g led to approximately 89.0% COD removal at the same time. The rise in the percentage of COD removal may be explained by an increase in IONPs dosage, which increases the number of active sites on the photo-catalyst surface; this in turn increases the number of hydroxyl radicals [15]. The results of this study are in agreement with the results obtained by Diya’uddeen et al. [30].

By assuming a pseudo-first-order reaction kinetics, Equation (3) was used to deduce the photocatalytic rate constants.

(3) $$-\frac{\mathrm{dCOD}}{\mathrm{dt}}=\mathrm{kCOD}↔\ln\left(\frac{{\mathrm{COD}}_{o}}{{\mathrm{COD}}_{t}}\right)=\mathrm{kt}$$

where COD_o_ and COD_t_ are the COD concentrations at time 0 and t, respectively and k is the pseudo first-order rate constant. The values of k were obtained from the slope of a plot of lnCOD_0_/COD_t_ vs. t. All the plots have high coefficient of regression values (Table 2), an indication that the removal of COD from synthetic petroleum wastewater by UV light and IONPs follows the pseudo-first-order kinetic model. The rate constants are listed in Table 2.

Table 2 also confirms that the increase in the time and IONPs dosages led to higher reaction rates. The rate constant for COD removal by UV light only was 0.0133 min^−1^, whereas, 0.0166 min^−1^, 0.0216 min^−1^, 0.0256 min^−1^, and 0.0269 min^−1^ were obtained for photocatalysis at IONPs dosage of 0.2 g, 0.4 g, 0.8 g, and 1.0 g, respectively. This shows that the photocatalytic degradation of synthetic petroleum wastewater is about 1.3–2.0 times faster in comparison to photolysis (UV light only). A total of 1.0 g of IONPs and 60 min irradiation time were kept constant and used to investigate the effect of pH.

#### 3.2.2. Effect of pH

**Figure 6:**
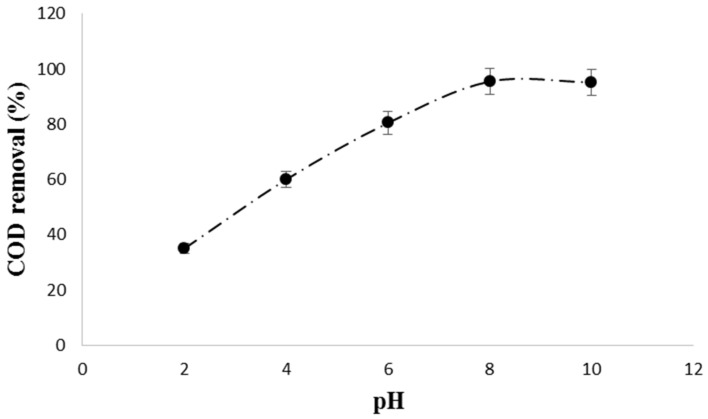
Effect of pH on COD removal from synthetic petroleum wastewater.

pH is an important parameter in photocatalysis because it affects the charge on catalyst particles, size of catalyst aggregates, and the positions of conductance and valence bands [31]. The results of this study showed that a maximum COD removal of 95.5% was achieved at pH 8 as shown in Figure 6.

**Figure 7:**
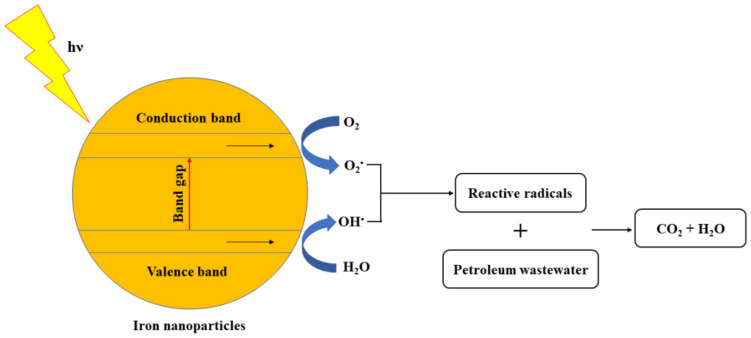
Scheme of the photocatalytic degradation of petroleum wastewater by IONPs/UV system.

The optimum pH of 8 is motivating for the IONPs/UV system since it falls within the pH range required for petroleum effluent discharge (pH 6–9). Moreover, the treatment system was able to lower the COD concentration from 1200 mg/L to 54.5 mg/L, an indication that the treated synthetic wastewater met the effluent discharge standards of 150–200 mg/L. Based on the results of this study, the COD and pH under investigation were reduced to levels below the minimum allowable limits for discharge into the receiving water bodies. A schematic mechanism of the degradation of petroleum wastewater by IONPs/UV system is presented in Figure 7.

In the IONPs, photogenerated holes in the valence band h_VB_^+^ react with H_2_O to generate OH^•^ species. Furthermore, e_CB_^-^ in the conduction band generate electron resonance plasma over IONPs surface and react with O_2_ to generate O_2_^•^. The reactive radicals generated (OH^•^ and O_2_^•^) react with the contaminants present in the wastewater to form CO_2_ and H_2_O. Thereby, resulting in the COD removal.

A comparison of COD removal from petroleum wastewater by various techniques with respect to IONPs/UV system is presented in Table 3.

The results obtained from this study show that the IONPs/UV system competes favourably with other techniques that has been reported in previous studies and hence, could be useful to lowering the COD (level of pollution) of petroleum wastewater.

## 4. Conclusions

Petroleum refineries discharge a large amount of wastewater during the refining process that contains hazardous constituents that are difficult to degrade. Given the complex and diverse nature of refinery wastewater pollutants, a combination of treatment methods is often the norm before reuse or discharge. In the present study, IONPs was synthesized from commercial reagents, characterized by modern instrumental techniques and was combine with UV light to degrade pollutants present in synthetic petroleum wastewater. The SEM and TEM micrographs of the IONPs show regular spherical shaped particles, the EDS, SAED, and XRF analyses confirm the synthesis of the nanoparticles, with mean particle size of 12.5 nm. For the IONPs/UV treatment system, the level of pollution of the synthetic petroleum wastewater was determined by the quantification of the COD. The treatment processes showed that the photocatalytic degradation efficiency of synthetic petroleum wastewater by the IONPs/UV system was strongly dependent on time, IONPs dosage and pH. At optimum conditions, COD removal efficacy of approximately 95.5% was achieved at pH of 8, 60 min exposure time, and 1.0 g IONPs dosage. The COD removal rates by a combined UV light and IONPs system were higher than UV light only, confirming a synergistic effect between UV light and IONPs. Therefore, the combination of UV light with IONPs could be beneficial for the degradation of petroleum refinery wastewater.

## Figures and Tables

**Table 1:** X-ray fluorescence spectrophotometry of iron oxide nanoparticles.

Species	Wt (%)
SiO_2_	29.50
Al_2_O_3_	2.16
Fe_2_O_3_	60.80
TiO_2_	0.19
CaO	0.12
P_2_O_5_	0.20
K_2_O	0.02
MnO	0.70
MgO	0.24
Na_2_O	0.32
Cu	0.05
Zn	ND
Rb	0.05
Zr	ND
Br	0.05
Cr	0.12
LOI	5.60
Total	100

**Table 2:** Pseudo-first-order rate constants.

Treatment Processes	Rate Equations	k (min^−1^)	R^2^
UV light only	y = 0.0133x + 0.1423	0.0133	0.9415
UV light + 0.2 g IONPs	y = 0.0166x + 0.2448	0.0166	0.9423
UV light + 0.4 g IONPs	y = 0.0216x + 0.3608	0.0216	0.9451
UV light + 0.8 g IONPs	y = 0.0256x + 0.4051	0.0256	0.9385
UV light + 1.0 g IONPs	y = 0.0269x + 0.5181	0.0269	0.9664

**Table 3:** Comparison of COD removal from petroleum wastewater by available techniques.

Processes	Time	pH	Max. COD Removal	References
TiO_2_/Sunlight	240 min	8.7	85%	[36]
Fenton−sequencing batch reactor	120 min	3	76.5%	[30]
Solar photo-two catalyst TiO_2_ and photo-Fenton	90 min	4.18	50%	[21]
Solar photocatalyst of TiO_2_/ZnO	170 min	6.8	76%	[31]
Electrocoagulation (EC)	40 min	7	99.1%	[37]
Solar photo-Fenton (H_2_O_2_/Fe^2+^/Solar)	180 min	3	78%	[38]
EC	60 min	8.5	94%	[24]
ZnO Photocatalyst	300 min	5	76%	
EC/ZnO Photocatalyst	180 min	-	95.8%	
UV/TiO_2_ UV/Zeolite	15 min	6	91% 92%	[23]
IONPs/UV system	60 min	8	95.5%	This study

## Data Availability

The data presented in this study are available on request from the corresponding author.
